# A Neural Network Approach to fMRI Binocular Visual Rivalry Task Analysis

**DOI:** 10.1371/journal.pone.0105206

**Published:** 2014-08-14

**Authors:** Nicola Bertolino, Stefania Ferraro, Anna Nigri, Maria Grazia Bruzzone, Francesco Ghielmetti, The Coma Research Centre (CRC) multidisciplinary team, on behalf of which the present publication was submitted, acknowledges the following members:, Matilde Leonardi, Eugenio Agostino Parati, Maria Grazia Bruzzone, Silvana Franceschetti, Dario Caldiroli, Davide Sattin, Ambra Giovannetti, Marco Pagani, Venusia Covelli, Francesca Ciaraffa, Jesus Vela Gomez, Barbara Reggiori, Stefania Ferraro, Anna Nigri, Ludovico D'Incerti, Ludovico Minati, Adrian Andronache, Cristina Rosazza, Patrik Fazio, Davide Rossi, Giulia Varotto, Ferruccio Panzica, Riccardo Benti, Giorgio Marotta, Franco Molteni

**Affiliations:** Scientific Director CRC; 1 Health Department, Carlo Besta Neurological Institute, Milan, Italy; 2 Neuro-Radiology Department, Carlo Besta Neurological Institute, Milan, Italy; University Of Cambridge, United Kingdom

## Abstract

The purpose of this study was to investigate whether artificial neural networks (ANN) are able to decode participants’ conscious experience perception from brain activity alone, using complex and ecological stimuli. To reach the aim we conducted pattern recognition data analysis on fMRI data acquired during the execution of a binocular visual rivalry paradigm (BR). Twelve healthy participants were submitted to fMRI during the execution of a binocular non-rivalry (BNR) and a BR paradigm in which two classes of stimuli (faces and houses) were presented. During the binocular rivalry paradigm, behavioral responses related to the switching between consciously perceived stimuli were also collected. First, we used the BNR paradigm as a functional localizer to identify the brain areas involved the processing of the stimuli. Second, we trained the ANN on the BNR fMRI data restricted to these regions of interest. Third, we applied the trained ANN to the BR data as a ‘brain reading’ tool to discriminate the pattern of neural activity between the two stimuli. Fourth, we verified the consistency of the ANN outputs with the collected behavioral indicators of which stimulus was consciously perceived by the participants. Our main results showed that the trained ANN was able to generalize across the two different tasks (i.e. BNR and BR) and to identify with high accuracy the cognitive state of the participants (i.e. which stimulus was consciously perceived) during the BR condition. The behavioral response, employed as control parameter, was compared with the network output and a statistically significant percentage of correspondences (p-value <0.05) were obtained for all subjects. In conclusion the present study provides a method based on multivariate pattern analysis to investigate the neural basis of visual consciousness during the BR phenomenon when behavioral indicators lack or are inconsistent, like in disorders of consciousness or sedated patients.

## Introduction

Multivariate pattern analysis (MVPA) is able to process information coming from differently located clusters of voxels and makes it possible to detect particular patterns of neural activity that may remain hidden to conventional analyses (e.g., univariate statistical methods) [1]. Indeed, in these last years, MVPA has been extensively applied as a “mind reading” tool to decode mental states from functional magnetic resonance imaging (fMRI) data, such as to assess perceptual states [2] or to evaluate deception and differentiate lying from truth-telling [3], [4], [5]. A great interest arose around fMRI studies using MVPA that allowed the investigation of how the contents of conscious experience are encoded in the brain [6].

Most of the work on this topic examined only the prediction of static and unchanging perceptual states during extended periods of stimulation [7], [8], [9].

A dynamic perceptual phenomenon particularly suitable to be studied with MVPA is the binocular visual rivalry (BR): two different visual stimuli are presented, one to each eye, and the two conflicting monocular images compete for access to consciousness and the subject usually experiences an alternate perception of the two images. The perceptual dominance of one image can endure for a few seconds before switching to the other, fluctuating stochastically over time [10], [11]. Thus, the visual input is the same, but the perceptual interpretation changes. Due to this characteristic, the BR paradigm was shown to be an important tool to explore the neural correlates of visual conscious experience [11], [12].

In this framework, Haynes et al. (2005) investigated BR using MVPA on fMRI signals [13]. They showed that linear discriminant analysis was able to predict in healthy subjects from brain activity alone the stream of visual consciousness by means of the fluctuation between two classes of simple stimuli (blue and red orthogonal rotating gratings). This study also demonstrated that accurate prediction of the perception during BR could be established with signals recorded during stable monocular viewing, suggesting the possibility to use this approach in the absence of behavioral indicators, such as in animals or patients with locked-in syndrome.

A seminal study by Tong et al. (1998) demonstrated that during BR in which houses and faces were presented, the fusiform face area (FFA) and the parahippocampal place area (PPA) reflected the perceived stimulus, showing that changes from house to face led to an increase in blood oxygen level dependent (BOLD) signal in FFA and a decrease in PPA, while changes from face to house led to the opposite pattern. Moreover they showed a striking resemblance of BOLD signal changes during non-rivalry and rivalry paradigms, not only in the qualitative pattern but also in the amplitude of FFA and PPA responses [14]. However, they did not test for generalization between training with non-rivalry, and testing with rivalry in absence of behavior.

The brain regions involved in the processing of these stimuli are the bilateral occipital area, collateral sulcus, PPA, occipital face area (OFA), and FFA. In particular FFA and OFA were identified as areas responding more to face stimuli, whereas bilateral PPA as more reactive to houses and objects [15], [16].

These results allowed us to investigate whether multivariate classification methods are able to decode a dynamic perception phenomenon of complex and ecological stimuli using rivalry and non-rivalry paradigms.

The aim of our study was to provide a method based on artificial neural networks (ANN) [17] able to identify the different neural pattern of activity related to the processing of two classes of visual stimuli (houses and faces) during a visual rivalry paradigm, applicable in the absence of behavioral indicators, indicating which stimulus is perceived by participant.

We studied 12 healthy subjects with fMRI as they viewed binocular non-rivalry (BNR) and BR tasks. First we used the BNR to identify brain areas involved in face and house decoding, then we trained the ANN on these data, and finally we employed the trained ANN in order to discriminate the pattern of activity in BR task analysis and verified the consistency of these results with the behavioral response.

A major challenge of this study was the signal decoding due to a low signal to noise ratio (SNR). Many system imperfections and physical phenomena (eddy currents, asymmetric anti-aliasing filter response, concomitant magnetic field, mismatched gradient group delays, and hysteresis) affected echo planar imaging (EPI), and especially sequences with short TR, by artifacts and signal loss [18]. Hence, a processing protocol for signal optimization was implemented in order to increase the network performance.

## Materials and Methods

### Participants

We recruited 12 healthy volunteers for this study (mean age 32.5 years, range 18–47 years) with no history of neurological disease, 5 of whom were female. The experimental protocol was approved by the ethics committee (Comitato Etico) of IRCCS Carlo Besta Neurological Institute and all the participants gave written informed consent. All clinical investigation has been conducted according to the principles expressed in the Declaration of Helsinki.

### MRI acquisitions

Anatomical and functional data were collected using a 3.0 Tesla MRI scanner (Achieva TX, Philips Medical Systems BV, Best, NL) equipped with a 32 channel phase-array head coil. Each participant underwent to an imaging protocol including anatomical 3D T1 (TFE with FOV = 240×240 mm^2^ and voxel = 1×1×1 mm^3^, TR/TE = 9.8/4.6 ms) and two EPI sequences, one for the BNR (200 volumes) and the other for the BR fMRI paradigm (600 volumes). Both fMRI sequences had a FOV = 240×240 mm^2^, an isotropic voxel (3×3×3 mm^3^), a 90° flip angle and a TE = 40 ms. The TR of the BNR-localizer sequence was 3000 ms, while for the rivalry sequence TR was 1000 ms. We chose a short TR of 1000 ms for the BR sequence in order to be sure to capture the rapid alternate perception between the two images. The perception dominance of one image was shown to be in the range between 2.5 to 5.5 s [14]. Because of the different TR, slices number of the package was set to 30 for the first EPI sequence and 16 for the second.

### fMRI paradigms

**Figure 1:**
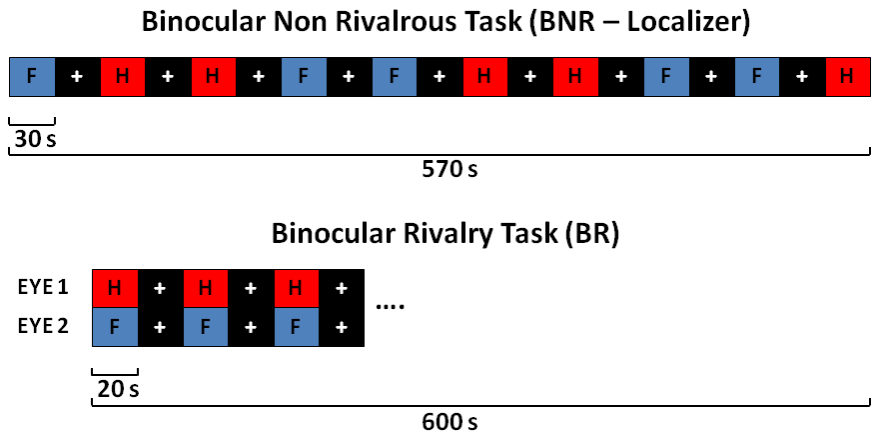
Diagrams showing the fMRI block tasks design. The letter H in the red box represents the house block, the letter F in the blue box represents the face block, and the white cross in the black box represents the rest block. The BNR task is shown on the top, while the BR task is shown on the bottom.

All participants performed two fMRI block design tasks (Fig. 1): the BNR-localizer and the BR paradigm. During the BNR-localizer task participants were presented with 5 blocks showing a set of faces alternating with 5 blocks showing a set of houses, spaced out by 10 rest blocks. Each block duration was 30 seconds and included 10 stimuli, each shown for 3 seconds.

During the BR task participants were presented with 15 picture blocks broken up by 15 rest blocks. For the picture blocks, a house was shown to one eye and a face was shown to the other simultaneously. These two pictures were chosen from those used for the BNR-localizer task. Each block duration was 20 seconds. The house and face images were presented to the right and left eye, respectively, for half of the participants, and vice versa for the other half. For both tasks a white fixation cross on a black background was presented during rest blocks. Additionally the house pictures were red-filtered while the face pictures were blue-filtered in order to employ stimuli similar to the ones used in the previous literature [13], [14] and to increase the perceptual differences between the two classes of stimuli.

All the participants were provided with a pair of stereo LCD goggles for visual stimulation, a pair of headphones, and two keypads (VisuaStim, Resonance Technology Inc., Northridge CA, USA). During the BR task participants were asked to indicate which picture they perceived by pressing a button on the keypad at transition points from one stimulus perception to the other, and behavioral data were collected.

### Data Analysis

**Figure 2:**
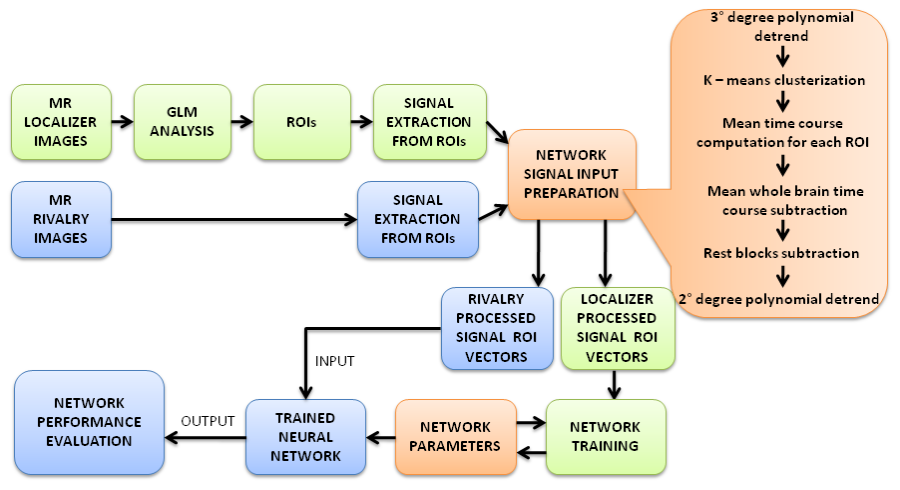
Diagram illustrating signal processing steps. In the green boxes the steps concerning the BNR ROI signals are described, in the blue boxes the steps concerning BR ROI signals are described, and in the orange boxes the steps in common for both signals are described.

In order to illustrate the multiple steps of the method employed, a flow chart is provided in Fig. 2.

For all the data analyses we used SPM 8 (Statistical Parametric Mapping, http://www.fil.ion.ucl.ac.uk), MatLab 7.13 (The MathWorks Inc., Natick, MA, 2012), and SPSS 17.0 (SPSS Inc., Chicago, 2008).

### Behavioral data analysis

During the BR task, the mean value of the duration of the perceptual dominance for each image (house or face) and its standard deviation were calculated for each participant and for the whole group.

### Data preprocessing

Both fMRI acquisitions (i.e., BNR and BR scans) were co-registered to the T1 and pre-processed to correct 3D motion artifacts, linear drifts, and low-frequency non linear drifts. Spatial smoothing was applied using a Gaussian kernel with a 5 mm full width at half maximum isotropic. For the BR scan, a slice timing correction was also performed.

### Single-subject analysis and ROIs identification

In order to identify the functional regions of interest (ROIs) (i.e., FFA, PPA, and OFA) necessary to extract the time course signals to train and to run the ANN we performed standard single-subject analyses [19] on the BNR-localizer data in the framework of the general linear model (GLM). In the design matrix we modeled the presentations of faces and houses as predictors. We performed two t-contrasts: faces>houses and houses>faces [14]. The package volume of the sequence, employed in the BR task, was applied as an inclusive mask to the obtained con-images in order to verify that the identified areas were included in the BR acquisition package and to control for I type error [20]. For every single subject the activations clusters, resulting from the analysis, were selected as ROIs with a voxel-level threshold of *P*<0.05 FWE-corrected and a minimum cluster size of 5 voxels. If using these threshold at least N = 3 ROIs (1 ROI for the first t-contrast and 2 ROIs for the second) were not identified, the voxel-level threshold was moved to p<0.05 FDR corrected. A maximum of 3 ROIs for contrast was extracted based on higher T-score. The peak MNI coordinates of every activation cluster, selected as ROI for each subject, is provided in Table 1.

**Table 1:** Selected ROIs coordinates.

	Faces-houses t-contrast												Houses-Faces t-contrast					
	FFA right			FFA left			OFA right			OFA left			PPA right			PPA left		
Subject #	x	y	z	x	y	z	x	y	z	x	y	z	x	y	z	x	y	z
1	40	−46	−22	−42	−60	−20	42	−68	−10	–	–	–	28	−46	−10	−28	−54	−8
2	40	−54	−16	–	–	–	–	–	–	–	–	–	30	−50	−8	−26	−50	−10
3	58	−28	16	−	–	–	–	–	–	–	–	–	36	−26	−12	−28	−36	−4
4	46	−48	−18	−42	−48	−20	–	–	–	–	–	–	32	−48	−10	−28	−50	−8
5	38	−44	−20	–	–	–	50	−68	−2	–	–	–	24	−42	−10	−26	−46	−12
6	44	−52	−22	–	–	–	40	−70	−10	–	–	–	32	−48	−10	−28	−56	−10
7	–	–	–	−44	−50	−24	38	−72	−20	−38	−78	−10	26	−50	−14	−24	−52	−14
8	40	−60	−18	–	–	–	–	–	–	–	–	–	30	−52	−8	−26	−44	−10
9	–	–	–	−38	−56	−18	44	−80	−18	–	–	–	28	−60	−6	−26	−52	−10
10	40	−40	−16	−36	−48	−14	–	–	–	–	–	–	28	−40	−8	−26	−44	−6

The table shows the peak MNI coordinates of every activation cluster, selected as ROI for each subject for PPAs, FFAs and OFAs.

### Network input signal preparation

For both BNR and BR datasets, we extracted the fMRI signal time-courses from each voxel in the identified N ROIs and the mean fMRI signal time-course from the whole brain. The ROIs and the mean whole brain signal time-courses obtained from both fMRI task scans were detrended with a third degree polynomial function to eliminate the signal drift. Afterwards, the k-means clusterization algorithm [21] was employed to split the BR detrended ROI signal time-courses in two clusters. Considering that the ROIs were selected by the analysis on BNR data, we employed the clusterization algorithm in order to discriminate the voxels participating in BR phenomenon and remove the voxels not involved in the activation pattern and also affected by higher noise. Then, the mean fMRI signal time-course of the remaining voxels of each ROI was calculated.

These signals and the mean fMRI time-course from the whole brain were converted in percent signal changes relative to their mean values over time. In order to minimize most of the non-task-related signal fluctuations, the percent signal changes of the whole brain fMRI signal time-course was subtracted from the percent signal changes of each single ROI [22]; the resulting signal was shifted to account for the BOLD response delay. Finally, we removed the rest block time points and detrended the signals with a second degree polynomial, obtaining the ultimate neural network input signal. In order to assess the reliability of the network output we used the removed rest block time points of the BR dataset as a control signal.

### Training and Running the network

We implemented a one-layer Feed-Forward Neural Network with a Log-Sigmoid Transfer Function [17]. We chose an hidden layer size of 65 neurons and, as performance function, the Mean Square Error (MSE) relative to the difference between the target outputs (presented stimuli) and the values predicted by the model (network outputs).

The network was trained and run separately for each subject. As a training dataset, we used a matrix in which the columns were the processed BNR ROIs time courses, divided randomly in train set (75%) and valuation set (25%). The training set was used for computing the gradient descent and updating the network weights and biases in the direction in which the performance function decreases more rapidly, while the evaluation set was used for the MSE value computation. At the end of the training, the network weights and biases were saved at the minimum of the MSE. If the training process performance did not achieve a selected threshold (MSE <0.02), chosen to have a good and homogeneous training between subjects [23], the algorithm repeated the process using new initialization seeds (weights and biases).

Next, we ran the trained network using the matrix in which the columns were the processed ROIs BR time courses as input data. The ANN produced an output matrix **X**, in which the rows were the time points and the two columns were the outputs of classification for the presented stimuli in a range between zero and one. The ideal output matrix row (1 0) represented the face, while (0 1) represented the house. In order to assign time points to one of the two conditions, we set up a threshold |X_t,1_– X_t,2_| >0.9, where t = 1,…,N, with N number of time points. If a time point did not reach the threshold, it was labeled as not assigned and discarded; the whole process, including training and run, was reiterated until the number of unassigned time points was smaller than 16.7%.

To evaluate the accuracy of the network to discriminate between perception status, we computed (1) the percentage of successes, obtained comparing the network output with the behavioral response vector, and (2) the p-value, obtained by using a binomial distribution, considering two conditions with a probability of 50% to be equal or different from behavioral responses.

### Assessment of reliability of the network output

As the network weights and biases change during initialization and optimization, the resulting output is affected by a certain variability, reflecting the stochastic nature of ANN training [23]. Hence, to evaluate the consistency of the results, for each subject we repeated the whole process described in the previous section 1000 times. In order to have a negative control set of data we applied the 1000 repetition again substituting the BR with the rest block time-course. For each repetition we collected the number of time points assigned to houses or faces. Based on the hypothesis that the rest block signal is unrelated to BR phenomenon, in order to highlight differences between the distribution of predicted stimuli (houses and faces), we analyzed the different outputs. Frequency histograms of houses and faces were produced and normality tests [24] were performed on the distribution of percentage value of the two stimuli over the total time points allocated along with mean, variance, kurtosis, and asymmetry computation. We expect that for participants who experienced the phenomenon, the event distribution in the BR-task signal ANN output is balanced between the two stimuli (i.e. ratio between the percentage of number of houses and faces >1/4) and leptokurtic or at the most normally distributed over the 1000 reiterations. We also collected evaluative information performing a comparison between the task and the rest block signal ANN output (control). This signal is expected to be characterized by a more asymmetric and/or platykurtic events distribution and/or an unbalanced distribution between stimuli, with a larger variance, because of the unpredictable random effects involved in the ANN time points attribution of a non-task-related signal.

## Results

We discarded two of the subjects from our analysis: the first because we did not find the expected activations (FFAs, OFAs, PPAs) during the GLM analysis of BNR paradigm, and the second because the subject declared that he did not experience the perception alternation phenomenon.

During the BR paradigm, the participants reported alternations between face-dominant and house-dominant percepts. The mean phase duration was 3.37 s (range: 1.78 to 4.47 s; standard deviation = 0.93 s).

**Figure 3:**
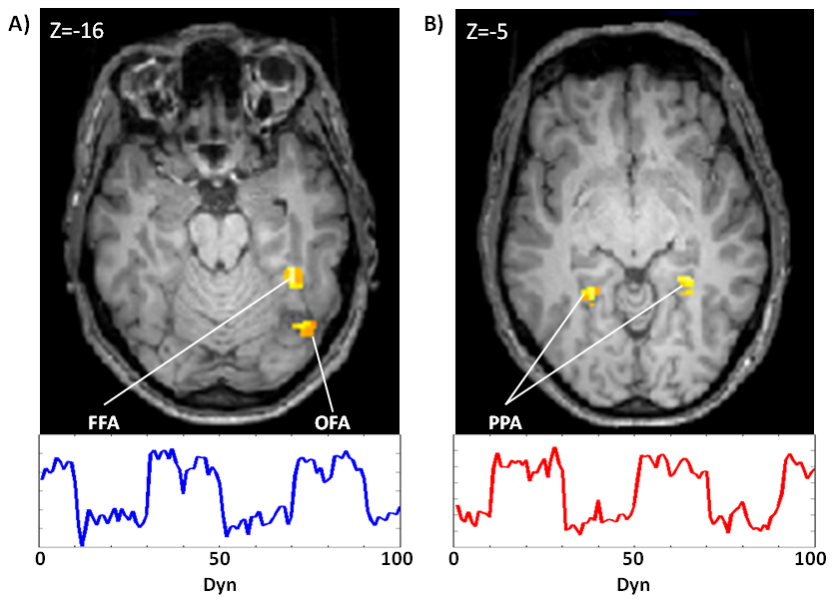
Example of resulting BOLD activity from GLM single-subject analysis of BNR-localizer task. Picture A shows t-contrast activations of face-house (FWE<0.05) in BNR time-course of PPA on top and FFA ROI on the bottom. Picture B shows t-contrast activations of house-face (FWE<0.05) in BNR-localizer time-course of FFA on top and PPA ROI on the bottom.

Consistent with the literature [15], [16], the single-subject analysis of BNR data revealed that the participants showed activity for the contrast faces>houses in the posterior fusiform gyrus (i.e., FFA) and in the inferior occipital gyrus (i.e., OFA) (Fig. 3A), while for the contrast houses>faces in the parahippocampal gyrus (Fig. 3B). We identified a maximum of 5 ROIs for 2 participants, 4 ROIs for 5 participants, and 3 ROIs for 3 participants (Table 1).

The mean number of signal time points not assigned to each of the two conditions was 10.7±3.7%.

**Figure 4:**
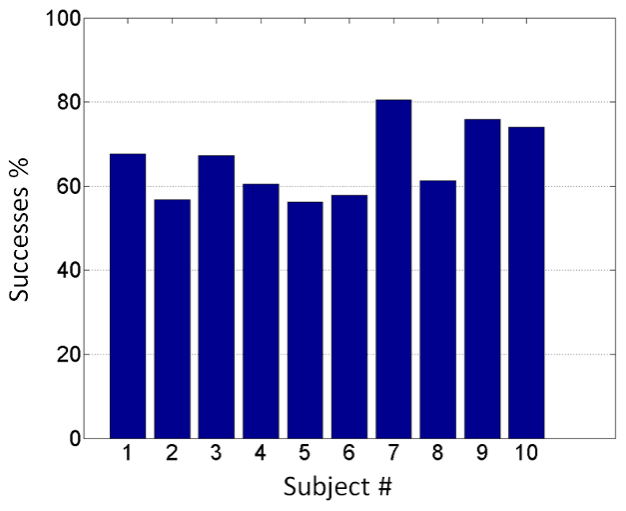
Bar plot of ANN percentage of successes for each subject. The plot shows in ordinate the percentage of time points correctly allocate to conditions (house or face) and in abscissa the number associated to the participants.

For 9 of the 10 participants, combined information of 3 ROIs was sufficient to allow the neural network to predict which stimulus the subject was experiencing with up to 75% accuracy (*p*<0.05). When the signals from 4 ROIs were combined the classification accuracy improved slightly for all but 1 participant, and the ANN predicted the perceived stimulus in all the participants (up to 78% accuracy; *p*<0.05). The only 2 participants in which 5 ROIs were detected showed a further slight increase of the accuracy of the neural network (up to 80%; p<0.05) combining the information coming from all of them (Table 2; Fig. 4).

**Table 2:** Results summary.

			3 ROI			4 ROI			5 ROI			Behavioral	
#	sex	age	Successes %	p	Descardedtrials (%)	Successes %	p	Descardedtrials (%)	Successes %	p	Descardedtrials (%)	mean (s)	SD (s)
1	M	32	**58,1**	0,004	6,7	**61,7**	0,000	8,7	**67,6**	0,000	12,6	3,13	1,28
2	M	47	**56,8**	0,016	10	–	–	–	–	–	–	2,89	1,28
3	F	24	**67,3**	0,000	13,3	–	–	–	–	–	–	2,97	1,08
4	M	35	**59,2**	0,000	10	**60,5**	0,000	8	–	–	–	4,47	1,6
5	M	40	**49,4**	0,603	16,7	**56,3**	0,026	10,3	–	–	–	2,57	1,58
6	M	17	**58,2**	0,005	9,7	**57,8**	0,009	14,3	–	–	–	1,78	0,76
7	F	40	**75,5**	0,000	1,1	**78,3**	0,000	13,7	**80,6**	0,000	12,6	4,35	2,03
8	M	37	**61,3**	0,000	9,7	–	–	–	–	–	–	3,97	2,4
9	F	23	**65,3**	0,000	15,3	**75,9**	0,000	14,3	–		–	2,94	2,5
10	M	32	**69,2**	0,000	2,6	**74,0**	0,000	6,3	–		–	4,6	3,15

Neural Network results obtained from all participants, making use of 3, 4, or 5 ROIs as input data for the algorithm. The rate of success increases with the number of ROIs employed.

**Figure 5:**
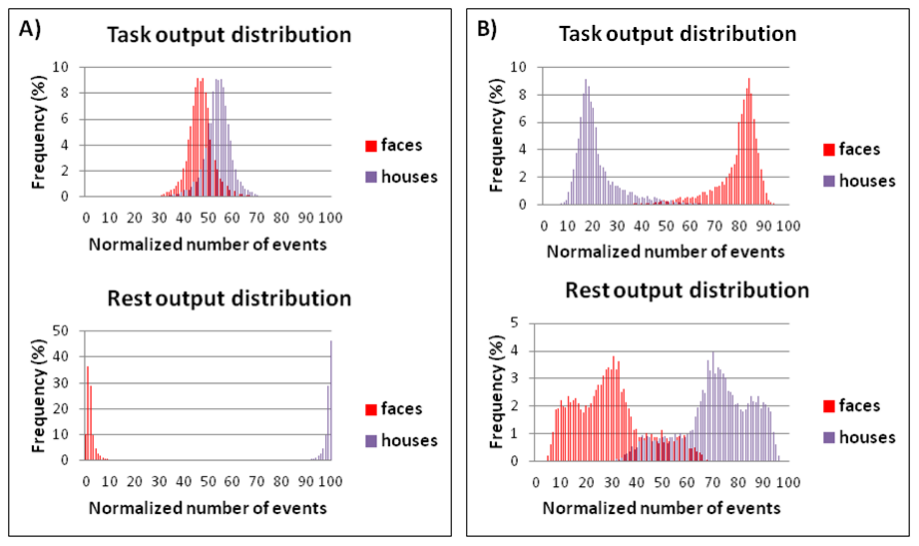
Examples of stimuli distributions after 1000 output repetitions. In panel A on top the bar histogram shows for subject 7 the results of the 1000 reiterations using the BR task time course signal. In y axis is represented the percentage number (frequency) of house and face over all time points in a reiteration, and in the x axis the percentage number of reiterations (events) in which we obtained a determined frequency of houses and faces over all 1000 reiterations. In the plot on the bottom of panel A the BR time course signal has been replaced with Rest time course signal. In panel B the same bar histograms, for BR and rest time courses, are shown for the subject excluded because he did not experience the BR phenomenon.

We tested the reliability of ANN output for all 10 participants, as described in the previous section. Analyzing the BR task signal, we found that in all cases the events distributions were leptokurtic (kurtosis coefficient >0) or normal (Shapiro-Wilk, p>0.05) and the mean number of predicted stimuli was balanced, with a percentage of houses and faces in the range between 25% and 75% for all the participants, except one. Moreover for the rest block signal output the events distribution was unbalanced and/or platykurtic (Kurtosis coefficient <0), with a larger variance and asymmetry than for task signal in all cases (Fig. 5A).

We also performed the reliability assessment for the discarded participant who declared that he did not experience the rivalry phenomenon. In this case the events distribution was unbalanced, platykurtic for both task and rest signal ANN output; moreover, asymmetry was larger for task than for rest ANN output (Fig. 5B).

## Discussion and Conclusions

The present study provides a method based on MVPA to investigate the neural basis of visual consciousness during the BR phenomenon when behavioral indicators, of what the participant is experiencing, are lacking or inconsistent.

Our main results showed that the trained ANN was able to generalize across two different fMRI paradigms (i.e. BNR and BR) and to identify with high accuracy the cognitive state of the participant (i.e. which stimulus was consciously perceived) during the BR condition. These results were obtained combining information from 3 ROIs in all the participants, except one, although the best performances were obtained by combining information from 4 or 5 ROIs. The possibility to apply this procedure using a limited number of ROIs makes it applicable to a wide variety of patients with cerebral insult.

Based on the literature, we decided to use faces and houses as stimuli because they induce very different patterns of neural activity supporting an optimal training of ANN and because faces, due to their ecological relevance, are easily discriminated than other stimuli [25].

The ability to identify a common pattern of neural activity during two different paradigms is extremely important in a context where behavioral indicators lack or are difficult to detect. As in the study by Haynes et al. [13], we trained the neural network with data obtained in a controlled condition (i.e., BNR condition) that allowed us to know what the participant was perceiving, and tested it on data obtained in a condition where there was no external control (except for the behavioral response, used only to assess the prediction accuracy of the algorithm) of what the individual was perceiving (i.e., the BR condition). This clearly supports the use of our ANN in conditions where the conscious perception of an individual is not accessible to an external observer. Unlike Haynes [13], who trained the neural network on data obtained from a monocular non-rivalry condition, we trained it on data obtained from a binocular non-rivalry condition. The ability of the ANN to predict the conscious perception during BR using a training on BNR fMRI signal indicates that the BOLD signal changes in the ROIs were strictly modulated by the conscious percept and not by the eye of origin of the stimuli.

The behavioral data showed that the mean perception dominance duration was variable between subjects and consistent with the previous studies using similar stimuli [14], although an EEG study reported a shorter perception dominance duration using rotating gratings as stimuli [26]. Interestingly, we noticed that the worst performance of our brain states classifier was obtained from subjects 5 and 6, who experienced shorter mean perception durations (respectively 2.57 s and 1.78 s); we speculate that it may be harder to decode a signal when perception changes are too quick.

The single-subject GLM analysis of the BNR task did not activate the same number of areas in all participants. Thus, it was not feasible to identify 4 or 5 ROIs for each subject, because OFA and FFA are functional areas and they do not correspond to an easily recognizable and circumscribed anatomical location [15]. For this reason we trained and tested the ANN using information extracted from 3, 4 and 5 ROIs, based on the available identified regions for each participant.

In order to create a non-user dependent standard procedure, two thresholds were fixed, one related to the network training performance (MSE <0.02) and the other to the time points assignment (|X_t,1_–X_t,2_| >0.9), based on the best results obtained for our group of healthy volunteers.

In the last decade there has been a great ongoing debate about the neural processes underlying BR, with some studies describing this phenomenon as a high-level and representation-based process [14], [27], [28], [29] and others describing it as a low–level and eye-based process [13], [30], [31]. In our study, we decided to use a paradigm based on the hypothesis of high-level and representation based processes during BR.

The future development of this study lies in the application of the described method to investigate BR phenomenon in patients with different levels of sedation, disorder of consciousness or patients with profound physical disabilities, where it is difficult even for experienced clinicians to diagnose cognitive ability [32]. The neuroscience community used fMRI paradigms extensively to detect willful behavior in these patients [33], [34].

The absence of any feedback from these patients creates the tricky problem of the ANN output truthfulness assessment.

We addressed this criticism by outlining some criteria that were derived from the described training method and reliability assessment. The conditions that should be fulfilled to consider the ANN output reliable and infer that the subject experienced the perception of BR, even in absence of any feedback, are as follows:

- activation clusters present in BNR task single-subject analysis necessary to identify at least 3 ROIs;
- high network training performance: MSE <0.02;
- after 1000 runs the BR-task signal ANN outputs must be balanced between stimuli and presenting a leptokurtic, or at most normal, events distribution;
- the BR-task signal ANN output events distribution must have a smaller variance and symmetry than the rest events distribution.

Despite the significance of the results obtained, we underline that the classification performances of the ANN could have been more accurate in consideration of some limits of our study. The main issues that cannot be easily resolved lies in the behavioral responses registration method and more specifically we identified three critical aspects: first we did not ask to the participants to inform us when the two percepts were overlapping, second subject reaction times and errors in button-pressing may alter our recorded data and bias our final estimation on ANN output and third during BNR task registration the motor behavioral responses were absent.

Another limit of this study may be linked to pulse sequences parameters used for BNR and BR scans: it would likely be possible to achieve a better performance of the network using the same EPI sequence parameters for both tasks, though differences between the two sequences are slight.

In conclusion, the present study provides a method based on multivariate pattern analysis to investigate the neural basis of visual consciousness during the BR phenomenon when behavioral indicators lack or are inconsistent.
